# Guiding principles and considerations for designing a well-structured curriculum for the brain-computer interface major based on the multidisciplinary nature of brain-computer interface

**DOI:** 10.3389/fnhum.2025.1554266

**Published:** 2025-05-15

**Authors:** Hengyuan Yang, Tianwen Li, Lei Zhao, Yanzhao Wei, Xiaogang Chen, Jiahui Pan, Yunfa Fu

**Affiliations:** ^1^Faculty of Information Engineering and Automation, Kunming University of Science and Technology, Kunming, China; ^2^Brain Cognition and Brain-Computer Intelligence Integration Group, Kunming University of Science and Technology, Kunming, China; ^3^Faculty of Science, Kunming University of Science and Technology, Kunming, China; ^4^Institute of Quantitative and Technological Economics, Chinese Academy of Social Sciences, Beijing, China; ^5^Institute of Biomedical Engineering, Chinese Academy of Medical Sciences and Peking Union Medical College, Tianjin, China; ^6^School of Artificial Intelligence, South China Normal University, Foshan, China

**Keywords:** brain-computer interface, BCI major, multidisciplinary nature of BCI, curriculum design, construction of BCI major

## Abstract

Brain-computer interface (BCI) is a novel human-computer interaction technology, and its rapid development has led to a growing demand for skilled BCI professionals, culminating in the emergence of the BCI major. Despite its significance, there is limited literature addressing the curriculum design for this emerging major. This paper seeks to bridge this gap by proposing and discussing a curricular framework for the BCI major, based on the inherently multidisciplinary nature of BCI research and development. The paper begins by elucidating the primary factors behind the emergence of the BCI major, the increasing demand for both medical and non-medical applications of BCI, and the corresponding need for specialized talent. It then delves into the multidisciplinary nature of BCI research and offers principles for curriculum design to address this nature. Based on these principles, the paper provides detailed suggestions for structuring a BCI curriculum. Finally, it discusses the challenges confronting the development of the BCI major, including the lack of consensus and international collaboration in the construction of the BCI major, as well as the inadequacy or lack of teaching materials. Future work needs to improve the curriculum design of the BCI major from a competency-oriented perspective. It is expected that this paper will provide a reference for the curriculum design and construction of the BCI major.

## 1 Introduction

**FIGURE 1:**
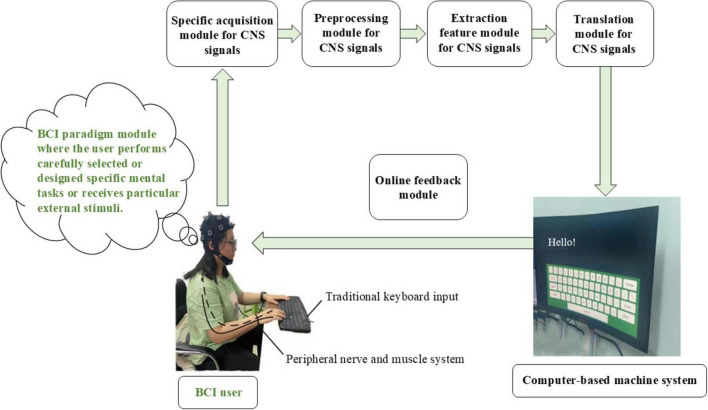
The schematic diagram for BCI system.

Brain-computer interface (BCI) is a revolutionizing human-computer interface (HCI) that bypasses or does not rely on peripheral nerves or muscles. Instead, it establishes a direct communication and control channel between the brain (or central nervous system, CNS) and computer using signals generated in the CNS (referred to as central nervous signals) (Allison et al., 2012; Graimann et al., 2010; Ramsey and Millán, 2020; Wolpaw and Wolpaw, 2012), as shown in Figure 1 (Chen et al., 2024; Luo et al., 2022). The goal of BCI is to improve or further enhance the quality of life or work efficiency of individuals, including patients, people with disabilities, and healthy individuals (Allison et al., 2012; Collura, 2014; Ramsey and Millán, 2020).

**FIGURE 2:**
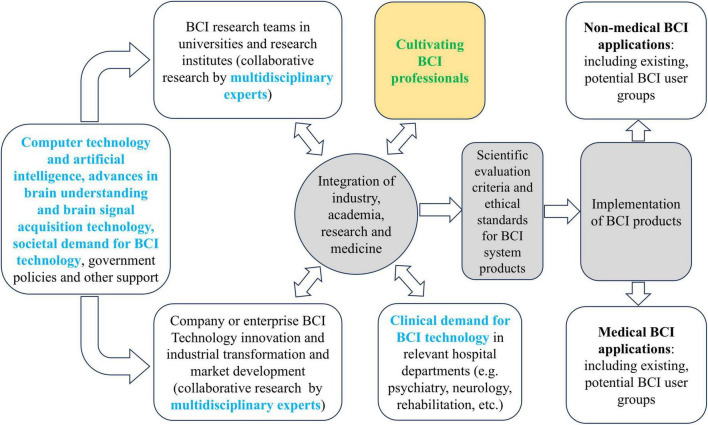
Demand for cultivating BCI professionals in the industrial transformation of BCI.

The rapid advances in computer technology and artificial intelligence, the progress in understanding the brain and acquiring brain signals, and the new understanding of the needs and capabilities of patients with neurological or psychiatric disorders and individuals with disabilities are major factors driving scientific interest and research activities in the BCI field (Wolpaw and Wolpaw, 2012). These factors have also driven the emergence of the BCI major. As BCI technologies develop rapidly and move toward industrial applications, the demand for trained BCI professionals has increased significantly, leading to an emerging BCI major, as illustrated in Figure 2 (Luo et al., 2022).

The BCI major is inherently multidisciplinary and requires extensive collaboration and integration across disciplines, unlike traditional or existing majors. Figure 2 also highlights the necessity for multidisciplinary cooperation in BCI research and applications. However, few publications have addressed the design of BCI curriculum based on its multidisciplinary nature. To fill this gap, this paper first discusses the emergence of the BCI major, followed by an explanation of the multidisciplinary nature of BCI research and development. The paper proposes principles for designing a BCI curriculum and suggests a proposed course structure based on this multidisciplinary nature. Finally, the paper discusses the challenges in the development of the BCI major and explores its prospects. It is anticipated that this paper will provide valuable suggestions for the BCI community regarding the curriculum design of the BCI major.

## 2 The emergence of the brain-computer interface major

### 2.1 The history of brain-computer interface research: an overview

BCI is a novel form of human-computer interaction, a new thing under the sun (Wolpaw and Wolpaw, 2012), and one of humanity’s dreams. Early research related to BCI is shown in Table 1.

**TABLE 1:** Early research related to BCI.

Years	Early research related to BCI
1924	Hans Berger discovered that electrical signals generated by the human brain could be recorded from the scalp. After 5 years of research, Berger published the initial of 14 papers that established the foundation for the utilization of electroencephalography (EEG) as a fundamental instrument for clinical diagnosis and brain research (İnce et al., 2021).
1938	In a 1938 holiday greeting from neurologist Herbert Jasper to Hans Berger included a diagram that was an early depiction of a brain-computer interface, suggesting that EEG signals could also be used in the realm of communication, albeit in a fanciful way (Wolpaw and Wolpaw, 2012).
1964	Gray Walter demonstrated EEG-based BCI. His seminal contributions to the field marked an early stage in the development of this technology (Walter et al., 1964).
1969 and 1971	Eberand Fetz and his colleagues demonstrated in the first neuron-based BCI that monkeys can learn to use individual cortical neurons to control meter pointers for food rewards (Fetz and Finocchio, 1971; Fetz, 1969).
1970s	The term “BCI” was first coined by Jacques Vidal in 1973 to denote any computer-based system capable of generating detailed information about brain function (Wolpaw and Wolpaw, 2012). The BCI system developed by Vidal utilizes visual evoked potential (VEP) recorded from the scalp above the visual cortex to determine the direction of the user’s eye gaze, thereby indicating the desired direction for cursor movement (Vidal, 1973).
Since the 1990s	In a research report in 1991, BCI was defined as a scientific term (Vidal, 1977). BCI has been clearly defined as a direct communication and control technique between the brain and computer systems since the 1990s. Initially, research in this domain was restricted to a few isolated laboratories. However, it had evolved into a vibrant and rapidly expanding scientific field by the early 21st century (Wolpaw and Wolpaw, 2012).
2012	The formal definition of a BCI, as established in 2012, stipulates that a BCI is a system that records CNS activity and converts it into an artificial output that replaces, restores, enhances, supplements, or improves the natural output of the CNS. Consequently, BCI modifies the way CNS interacts with the rest of the body or the external world (Blankertz et al., 2007; Daly and Wolpaw, 2008; Donoghue, 2002; Graimann et al., 2010; Schwartz et al., 2004; Wolpaw et al., 1991).

In Table 1, the BCI system developed by Vidal aligns with the modern, narrow definition of BCI (Wolpaw and Wolpaw, 2012). Contemporary VEP-based BCI still fundamentally builds upon this idea (Chen et al., 2024). The possibility of using signals recorded from the brain for communication and control gained traction and scientific interest in the decades leading up to 2011 (Birbaumer et al., 1999; Chapin et al., 1999; Millan et al., 2004; Musallam et al., 2004; Nicolelis, 2001; Taylor et al., 2002). However, sustained research only began in the 25 years between 1986 and 2011, and a recognizable field of BCI research and development emerged in the 15 years between 1996 and 2011 (Wolpaw and Wolpaw, 2012). By 2011, this emerging field had already seen several hundred research groups established worldwide, with new groups continuing to emerge (Wolpaw and Wolpaw, 2012). The development of the BCI field has been extraordinary, with 2006–2011 marking a “golden era” of BCI research, during which many seminal BCI studies were published (Daly and Wolpaw, 2008; Hochberg et al., 2006; LaConte, 2011; Santhanam et al., 2006; Scott, 2006). Since 2012, there has been a significant surge in BCI-related publications in prestigious international journals (Aflalo et al., 2015; Coulter and Kemere, 2023; Flesher et al., 2021; Hochberg et al., 2012; Lai et al., 2023; Metzger et al., 2023; Minev et al., 2015; Ramsey and Crone, 2023; Willett et al., 2021; Willett et al., 2023). Notably, the field has seen accelerated progress since 2015, when the BCI community established the EU roadmap for BCI development. This momentum has been further strengthened by investments from major companies, such as Neuralink, which have contributed to the advancement of BCI research and development. As previously stated, while most BCI research has focused on non-invasive approaches, invasive BCI has also undergone significant advancements since early studies first demonstrated the feasibility of directly linking neural activity to external devices. Pioneering work by Lebedev and Nicolelis (2006) and Nicolelis (2003) laid the foundation for invasive BCIs by demonstrating their ability to control external devices through animal models and preliminary human trials. Currently, invasive BCI research is focused on continuous optimization, with an emphasis on improving signal quality, system stability, and long-term biocompatibility to support clinical applications such as precise control of prostheses, wheelchairs, and communication devices. Recent studies (Lorach et al., 2023; Metzger et al., 2023; Willett et al., 2021) have further enhanced system safety and performance by integrating advanced signal processing techniques, deep learning algorithms, and novel biomaterials, paving the way for high-resolution, closed-loop control systems. In the future, invasive BCIs are expected to enable functional restoration, neurorehabilitation, and cognitive enhancement, potentially driving revolutionary breakthroughs in neuroscience and rehabilitation medicine.

In summary, the field has evolved over more than 60 years since the pioneering BCI research of Walter et al. (1964). The accumulated research achievements indicate that humanity is steadily moving closer to realizing the dream of BCI. The emergence of the BCI major and its associated talent cultivation will further drive the realization of this vision.

### 2.2 Key factors in the emergence of the brain-computer interface major

The significant surge in scientific interest and research activities in the field of BCI can be attributed to the convergence of the following three factors (Wolpaw and Wolpaw, 2012), which are also the key factors for the emergence of the BCI major (Yang et al., 2024).

The first and most apparent factor is the availability of powerful and affordable computer hardware and software capable of supporting the complex and high-speed analysis of brain activity that is essential for real-time BCI operation (Wolpaw and Wolpaw, 2012).

The second factor lies in the advancements over the past 60 years in understanding CNS through studies on both animals and humans. These efforts have yielded a wealth of new information about the nature and functionality of brain signals, such as EEG activity and neuronal action potentials (Wolpaw and Wolpaw, 2012). Simultaneously, significant advances have been made in brain signal recording technologies. Revolutionary breakthroughs have been achieved in understanding the extraordinary adaptability and plasticity of the brain and CNS. Harnessing these adaptive properties has made it possible to create novel interactions between the brain and computer-based devices. These interactions can replace, restore, enhance, supplement, or improve the natural interactions between the brain and its internal and external environments, a profoundly exciting prospect (Wolpaw and Wolpaw, 2012).

The third factor is the growing recognition of the needs and capabilities of individuals with disabilities. These disabilities often result from conditions such as cerebral palsy, spinal cord injury, stroke, amyotrophic lateral sclerosis (ALS), multiple sclerosis, and muscular dystrophy. Technologies such as home ventilators and other life-support systems now allow the most severely disabled individuals to live for many years. However, some of these individuals experience a significantly reduced quality of life due to difficulties in communicating with the outside world. Providing even the most basic means of communication and control to individuals with minimal voluntary muscle control could enable them to lead enjoyable and productive lives, which is a central goal of BCI. Even in its current limited state of development, BCI can also address this critical need (Wolpaw and Wolpaw, 2012).

The societal demand for BCI serves as a fundamental driving force behind its development. The construction of the BCI major and the refinement of its curriculum may be urgently needed to meet the growing demand for skilled professionals in BCI research, development, and applications (Yang et al., 2024).

### 2.3 Medical and non-medical application demands for brain-computer interface

The medical and non-medical application demands of BCI are the fundamental driving forces behind the emergence of the BCI major (Yang et al., 2024).

#### 2.3.1 Medical application demands for brain-computer interface

Patients with severe motor dysfunctions often lose the ability to communicate with the external world, directly reducing their quality of life and placing a heavy burden on their families and society. BCI can provide these patients with critical new opportunities for communication and control. As previously mentioned, this is the most important and primary goal of BCI (Flesher et al., 2021; Hochberg et al., 2012; Lai et al., 2023; Metzger et al., 2023; Ramsey and Crone, 2023; Willett et al., 2021; Willett et al., 2023). Other severely disabled patients also have potential needs for BCI technology, such as those with severe neurological and psychiatric disorders including spinal cord injury, stroke, Alzheimer’s disease, Parkinson’s disease, depression, and schizophrenia (Ramsey and Millán, 2020).

Numerous studies have demonstrated that BCI can replace, restore, enhance, supplement, or improve the natural output of the CNS for clinical patients and individuals with disabilities (Wolpaw and Wolpaw, 2012). For instance, BCI has been applied in stroke rehabilitation, the treatment of ALS, brain injury therapy, spinal cord injury rehabilitation, communication for locked-in syndrome patients, control of robots and prosthetics, neurorehabilitation training, enhancement of brain plasticity and attentional control, consciousness assessment and communication for patients with severe brain injury, and intelligent neuromodulation for movement disorders (Ramsey and Millán, 2020). In addition, research has explored the use of bidirectional BCI technology for spinal cord injury patients, the integration of BCI with virtual reality (VR) for neurorehabilitation, and the combination of BCI with functional electrical stimulation (FES) for motor recovery (Ramsey and Millán, 2020). Finally, as wearable neurotechnology, BCI holds promise for self-health monitoring to prevent diseases, representing a preventive medical application of BCI (Ramsey and Millán, 2020).

#### 2.3.2 Non-medical application demands for brain-computer interface

In addition to medical applications, BCI also has non-medical application demands or objectives. With the advancement of BCI technology, its non-medical applications are also growing, including applications in virtual reality (VR) and augmented reality (AR) (Kohli et al., 2022), gaming and entertainment (Prapas et al., 2023; Vasiljevic et al., 2024), education and learning (Jamil et al., 2021), as well as occupational safety and productivity enhancement (Al-Hudhud et al., 2019; Powers et al., 2015).

### 2.4 Demand for brain-computer interface professionals

BCI research, development and applications require BCI professionals to realize the human dream of BCI and to meet the demand for BCI medical and non-medical applications. With the rapid development of BCI technology and the acceleration of its industrial transformation, the demand for BCI professionals has increased significantly. The key factors driving this demand include: (1) The BCI industry’s need for interdisciplinary and innovative talent. (2) The growing application demands and industrialization of BCI require skilled professionals. (3) The need for experts capable of analyzing and interpreting complex brain signals. (4) The potential of BCI in future intelligent systems, which further emphasizes the importance of professional expertise (Yang et al., 2024).

As previously mentioned, the rapid development of BCI technology and its translation into industrial contexts has led to the emergence of an emerging major: the BCI major. The establishment of this major, in conjunction with the refinement of its curriculum and the formulation of training programs, appears imperative to meet the growing demand for BCI professionals in research, development and applications (Yang et al., 2024). The BCI major is distinguished by its inherent interdisciplinary character, necessitating extensive collaboration and integration across multiple fields to nurture talent that can make innovative and original contributions.

## 3 Multidisciplinary nature of brain-computer interface research and development and curriculum design of the brain-computer interface major

### 3.1 Multidisciplinary nature of brain-computer interface research and development

Since 2000, BCI has achieved remarkable progress, largely due to its inherent and indispensable multidisciplinary nature, which also defines the uniqueness of BCI research and development (Wolpaw and Wolpaw, 2012). Figure 1 illustrates the operational sequence from the user’s brain to the BCI, clearly demonstrating this characteristic. Table 2 lists the main disciplines involved in BCI research and development (Wolpaw and Wolpaw, 2012; Yang et al., 2024).

**TABLE 2:** Main disciplines involved in BCI research and development.

Number	Main disciplines involved	Brief description
1	Basic neuroscience, applied neuroscience, psychology, and cognitive science	The selection of brain signals for use in a BCI is contingent upon a comprehensive understanding of neuroscience, encompassing both basic and applied domains. The design and neural coding of the BCI paradigm are further influenced by the principles of psychology and cognitive neuroscience.
2	Physics, electrical engineering, materials engineering, neurosurgery, histobiology, and weak biosignal recording	The accurate recording of specific brain signals is contingent on the contributions of multiple disciplines, including the physical sciences, electrical and materials engineering, as well as neurosurgery and histobiology. Among other things, techniques for recording weak biological signals, including brain signals, play a crucial and fundamental role in the development of BCI.
3	Applied mathematics, computer science and technology, and digital signal processing	The appropriate, efficient, and timely processing of recorded brain signals necessitates the integration of computer science, engineering, applied mathematics, and signal processing techniques.
4	Algorithm analysis and design, pattern recognition, machine learning, deep learning and advanced artificial intelligence, systems engineering, and neuroplasticity	The design and operation of algorithms to translate brain signal features into device commands that implement user intent depends on systems engineering and an understanding of spontaneous and adaptive changes in brain function (i.e., brain adaptivity or neuroplasticity). The recognition of user intent from brain signals necessitates pattern recognition, machine learning, deep learning, and advanced artificial intelligence.
5	Clinical neurology, rehabilitation engineering, neuroengineering, biomedical engineering, and assistive technology	The selection of appropriate user groups with the realization of appropriate application systems requires clinical neurology and rehabilitation engineering, as well as neuroengineering and biomedical engineering, and relies on expertise in assistive technology.
6	Behavioral psychology, ergonomics, and human-computer interaction	The management of intricate, persistent interactions among users and application devices necessitates a comprehensive grasp of behavioral psychology and ergonomics. Secondly, BCI, as a novel form of human-computer interaction, necessitates a comprehensive grasp of the principles and methodologies that underpin human-computer interaction in general.

**FIGURE 3:**
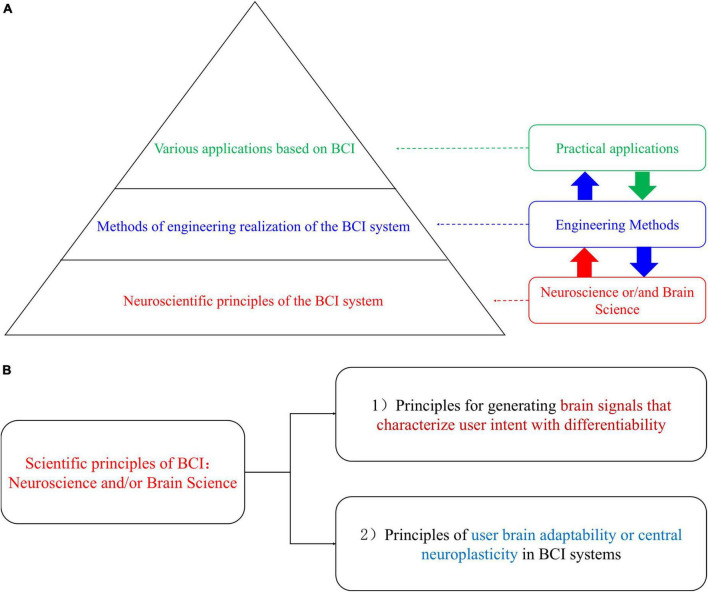
Three important aspects are involved in BCI research and development. **(A)** The relationship between neuroscientific principles, engineering methods of realization, and practical applications in BCI research and development; **(B)** the scientific principles of BCI.

Table 2 shows that the development of BCI has been quite different from previous or existing technologies. It requires the collaboration of multiple disciplines to create a successful BCI system. In particular, BCI research and development are directly linked to the brain that a highly complex system. This connection is the most distinctive and challenging aspect of BCI development. Neuroscience or Brain Science serves as the foundation for the scientific principles of BCI. Brain science includes areas such as central nervous system plasticity, psychological science, and cognitive neuroscience. It is particularly important to emphasize that the development of BCI is not merely an engineering challenge involving the implementation and optimization of BCI systems. Instead, it also relies on more fundamental and critical scientific principles, as shown in Figure 3A. Figure 3B illustrates the scientific principles underlying BCI systems. These principles involve identifying a specific set of psychological tasks, attentional tasks, and external stimuli that elicit highly separable brain signals for BCI applications. This is reflected in BCI paradigms and neural encoding (Tai et al., 2024). Additionally, the adaptability of the user’s brain, or central nervous system plasticity, plays a critical role in BCI systems. It serves as the scientific basis for modulating users’ neural activity and adapting BCI algorithms.

**FIGURE 4:**
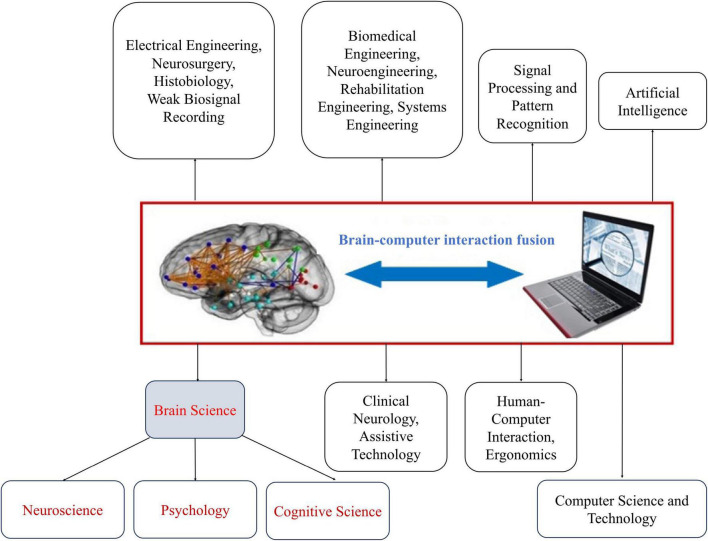
The schematic for the multidisciplinary nature of BCI research and development.

In addition to being directly related to neuroscience or brain science, BCI is also closely related to advanced computer science and technology, and artificial intelligence. This includes areas such as computer programming, software engineering, algorithm analysis and design, modern signal processing, pattern recognition, machine learning, deep learning, and artificial intelligence (Yang et al., 2024). Computer science and AI serve as tools or engineering methods for the implementation of BCI systems. In addition, user-centered BCI design is directly associated with human ergonomics (Lu et al., 2021) and is also closely related to fields such as biomedical engineering, neural and rehabilitation engineering, and intelligent robotic control. Figure 4 illustrates the multidisciplinary nature of BCI research and development (Yang et al., 2024).

In conclusion, given the multidisciplinary and unique nature of BCI research and development (particularly its direct connection with the brain). Effective collaboration among these diverse disciplines is essential for achieving the primary goal of BCI: providing significant new communication and control options for individuals with severe disabilities (Wolpaw and Wolpaw, 2012). Effective interdisciplinary collaboration is also required to achieve other goals of BCI beyond the primary goal (e.g., additional medical or non-medical applications) (Yang et al., 2024).

### 3.2 Principles of curriculum design for the brain-computer interface major

As mentioned above, BCI research and development is inherently multidisciplinary. Therefore, the curriculum design of the BCI major must fully consider its multidisciplinary characteristics and integrate different knowledge systems and practical methodologies. In this unique context, the following eight curriculum design principles are proposed to effectively construct the BCI major.

#### 3.2.1 Multidisciplinary collaboration based on brain-computer interface research and development

As shown in Figure 4, BCI system development involves numerous disciplines. Successful development of a BCI system (i.e., one that provides high usability and user satisfaction) requires multidisciplinary collaboration. The curriculum should strive to include essential disciplines in the course structure. Consideration should also be given to additional disciplinary support in the curriculum if needed.

#### 3.2.2 Content selection and necessity principle

Given the wide range of disciplines involved in BCI, each containing extensive content, it is essential to prioritize the knowledge most relevant to BCI system development. This means not simply including all knowledge from related disciplines in the curriculum. For example, careful consideration must be given to which aspects of neuroscience, psychology, and cognitive science are essential for BCI education, as well as which specific content from computer science and artificial intelligence should be integrated into the curriculum.

#### 3.2.3 Fostering interdisciplinary innovation as a core principle

BCI is a fundamental driver of innovation as a revolutionizing human-computer interaction technology. The curriculum should focus on cultivating students’ ability to innovate across disciplines. Modules such as thematic seminars, open-ended experiments, and capstone projects can be incorporated to inspire students’ innovative thinking and encourage interdisciplinary research and technological exploration.

#### 3.2.4 Integration of brain-computer interface principles, practices, and project-based learning

The curriculum should embed practical BCI development projects into the coursework, in addition to teaching the theoretical principles of BCI. This can be achieved through BCI experiments, training, internships, and research projects. Collaborating with BCI companies on projects can provide students with real-world scenarios to apply their theoretical knowledge. This project-based learning approach increases students’ interest in learning and their ability to address practical challenges in BCI development. It also helps them develop teamwork skills and the ability to make decisions under uncertain conditions.

#### 3.2.5 Flexibility and dynamic updates in curriculum design

Several seminal works in the field of BCI have been published (Allison et al., 2012; Graimann et al., 2010; Ramsey and Millán, 2020; Wolpaw and Wolpaw, 2012), and these publications serve as essential teaching references for senior undergraduate and graduate students at various universities and research institutes worldwide, including in China. They provide critical support for the curriculum design of the BCI major, enabling students to systematically master the underlying principles and practical applications of BCI technology. However, as BCI technology and its associated disciplines continue to evolve, the curriculum must remain flexible and be periodically updated. For instance, it is imperative to promptly incorporate the latest advances in neuroscience, psychology, and cognitive science, as well as emerging engineering methods and application cases, into the curriculum. Extensive exchanges within the BCI community facilitate this process, allowing both educators and students to stay current with the latest developments. For example, master classes of the tenth international brain-computer interface meeting in 2023 (Cernera et al., 2025) showcased recent research outcomes across diverse areas—from signal acquisition and decoding to system implementation—thus fostering interdisciplinary interaction and providing cutting-edge insights and practical experience to further advance BCI applications in clinical rehabilitation, assistive communication, and intelligent control. This dynamic exchange platform not only enables students to keep pace with evolving technologies and receive expert feedback on their research but also establishes a solid foundation for the continual update and improvement of the BCI major curriculum.

#### 3.2.6 Fostering self-directed learning and critical thinking

Adhering to a student-centered approach that values individuality, the curriculum should emphasize cultivating independent thinking, free inquiry, and questioning scientific spirit in the learning and research process. To achieve this, students’ ability to learn in a self-directed manner can be enhanced through flexible elective modules and personalized learning plans. Additionally, course content and teaching methods should encourage students to question and critically evaluate existing results or conclusions, and foster their ability to propose and test new ideas.

#### 3.2.7 Humanities and social sciences literacy

In addition to the interdisciplinary nature of BCI, students in the BCI major are expected to develop literacy in the humanities and social sciences. They are encouraged to take courses either related to or entirely different from their major to broaden their perspectives. Because of the significant social impact of BCI, the curriculum must include education in ethics and social responsibility. Students should understand the challenges posed by BCI in areas such as mental and physical health, privacy, and the misuse of technology, and cultivate a strong sense of social responsibility.

#### 3.2.8 Assessment based on learning processes and outcomes

Student assessment should go beyond final exam scores to include diverse methods such as class participation, group collaboration, research reports, and practical performance. This multidimensional assessment approach emphasizes a comprehensive evaluation of students’ learning processes and abilities.

By implementing these principles, the BCI curriculum can meet the demands of a multidisciplinary educational environment and support the development of students into innovative and practice-oriented interdisciplinary professionals.

### 3.3 Curriculum design for the brain-computer interface major

**FIGURE 5:**
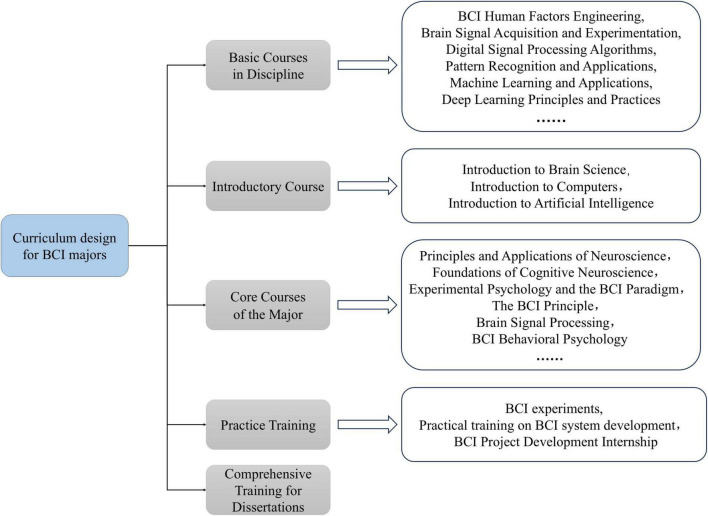
Proposed hierarchical structure of curriculum design for the BCI major.

It is recommended that the curriculum design for the BCI major consider the principles to establish a structured and well-organized knowledge system. This system could include disciplinary basic courses, introductory courses (including brain science, computer science, and artificial intelligence), core courses of the major, practice training for the major (including BCI experiments, training, and internships), and comprehensive training for the dissertation, as illustrated in Figure 5. Through systematic progression from basic courses to specialized courses and then to practical courses, students can gradually deepen their understanding of BCI technology during their studies.

In Figure 5, the inclusion of introductory courses for major disciplines ensures that students gain a broad understanding of brain science, computer science, and artificial intelligence as they relate to BCI development. This fosters research interest and lays the foundation for subsequent courses. The integration of practice training helps students combine BCI theories, principles, and methods with practice experience, thereby enhancing their understanding of specialized BCI course content. This also improves their ability to solve practical problems and nurtures innovation in real-world BCI application scenarios. The inclusion of comprehensive training for the dissertation component aids in developing students’ mastery of key skills such as BCI research methodologies, data analysis, and results interpretation. This process enhances their innovation capacity and critical thinking abilities.

In the practical teaching of the BCI major, phased objectives, project-based tasks, and theoretical integration are combined to enhance students’ research capabilities and problem-solving skills effectively. Practical training is structured into three stages. the foundational skills phase, where students use EEG devices to acquire and preprocess motor imagery-related brain signals while mastering noise filtering and feature extraction techniques; the system development phase, exemplified by the creation of a P300-based speller system; and the application innovation phase, where VR technology is integrated to develop neurorehabilitation training systems, fostering interdisciplinary collaboration and real-world application in complex scenarios. Practical projects encompass classical paradigms such as steady-state visual evoked potentials (SSVEP) and MI-BCI, advanced research topics (e.g., invasive BCI signal acquisition and deep learning-based decoding), and interdisciplinary applications (e.g., brain-controlled robotics and AR-BCI game development). Regarding resource utilization, open-source hardware such as OpenBCI and virtual simulation platforms are employed to reduce experimental costs. Additionally, a multifaceted evaluation framework—including project defenses, user testing, and competitive events—is implemented to comprehensively assess students’ technical proficiency, innovative thinking, and teamwork abilities.

In Figure 5, the inclusion of disciplinary basic courses aims to meet the demands of interdisciplinary collaboration and integration inherent in BCI development, laying a solid foundation for future research, development, and application in the field. Table 3 lists the proposed disciplinary basic courses for the BCI major and their necessity (Yang et al., 2024). These courses include essential mathematical foundations for quantitative analysis of brain signals and system modeling in BCI system development, such as calculus, linear algebra, and probability and statistics. Additionally, courses supporting BCI hardware development are included, such as physics and brain signal acquisition technologies. Furthermore, the curriculum also includes foundational courses for BCI software development, such as algorithm analysis and design, digital signal processing algorithms, and data structures, which are also part of the curriculum.

**TABLE 3:** Proposed basic courses in the discipline for the BCI major.

Course	Necessity
Calculus	Provide mathematical tools for the dynamic modeling of BCI systems (e.g., time series analysis of neural signals) and optimization algorithms (e.g., gradient descent). [0.7pt]
Linear algebra	Support matrix operations in machine learning models (e.g., dimensionality reduction via principal component analysis), directly impacting the efficiency of brain signal feature extraction. [0.7pt]
Physics and experiments	Help students understand the principles of electromagnetic signal acquisition (e.g., the physical mechanism of EEG devices), laying the foundation for designing low-noise brain signal acquisition hardware.
Advanced linear algebra	Fundamentals of BCI modeling, data processing, machine learning, computational intelligence, etc.
Probability and mathematical statistics	Provide a basis for statistical analysis of BCI data, thereby facilitating the modeling of uncertainty.
Algorithm analysis and design	The speed and efficiency of brain signal processing and decoding algorithms in BCI systems are crucial. The design of effective algorithms can lead to substantial improvements in the performance of BCI systems.
Foundations and experiments of analog and digital electronics	The hardware foundation of BCI is designed to facilitate the acquisition and processing of brain signals.
Discrete mathematics	Mathematical foundations of BCI and computers.
Human factors engineering of BCI	The enhancement of the user experience and satisfaction with the BCI system can be achieved through the implementation of user-centered design and evaluation.
Mathematical experiments	Lay the foundation for improving students’ BCI system modeling, data processing, experimental design, and implementation skills.
Brain signal acquisition techniques and experiments	Brain signal acquisition technology is a core module of BCI to develop student’s practical skills.
Neurosurgery and histobiology	Understand the surgical procedures for implantable BCI, the biological and medical basis for implanted devices or materials, and the properties of brain tissue.
Digital signal processing algorithm	Learn techniques such as filtering and Fourier transform, directly applying them to eliminate power line interference in brain signals and improve decoding accuracy.
Computer and system architecture	Provide support for computer technology in BCI systems, to understand the fundamentals of computer systems, and support the design and optimization of BCI systems.
Signals and systems	Provide students with basic signal processing concepts and methods for designing and optimizing BCI systems.
Data structure	The BCI system involves processing and real-time computation of various types of data. Mastery of the data structure facilitates more effective management and processing of the data, thereby optimizing the efficiency of the algorithms.
Stochastic processes and advanced statistical analysis	Improve BCI data analysis and support BCI system modeling and prediction by understanding the complex stochastic phenomena of brain and CNS signals.
Pattern recognition and applications	From an engineering perspective, BCI system is a pattern recognition system, an application of pattern recognition principles and methods.
Object-oriented programming and software engineering	An important component of the BCI system is the software system, and this course provides a foundation for the design, implementation, and maintenance of the BCI system.
New advanced artificial intelligence	The rapid development of new and advanced AI technologies can enhance the intelligence of BCI systems and provide innovative approaches to their development.
Virtual and augmented reality technology	BCI system utility is closely related to user experience and satisfaction, VR and AR technology can provide vivid images or intuitive immersive interactive environments to improve user experience and satisfaction.
Machine learning and applications	Methods of neural decoding for BCI systems are typically based on machine learning, which is a key technology for BCI.
Principles and practices of deep learning	The utilization of deep learning algorithms holds great promise in enhancing the efficacy of decoding complex intents in BCI systems.
Clinical neuropsychiatry	An in-depth understanding of the pathogenesis and rehabilitation needs of neuropsychiatric disorders can help design and implement BCI systems more effectively and enhance treatment outcomes.
Systems engineering	BCI systems are typically complex, multifaceted systems. Understanding systems engineering principles and methods helps to optimize performance by considering BCI system components and interrelationships during design.
Ethics for BCI	Popularize BCI-related ethical research and applications among students.

The inclusion of specialized core courses in Figure 5 helps students gain a solid understanding of the fundamental principles and practical skills specific to the BCI field. Table 4 outlines the proposed core courses for the BCI major and their necessity (Yang et al., 2024). These courses include principles fundamental to BCI systems, such as BCI principles, neuroscience principles and applications, and fundamentals of cognitive neuroscience. Additionally, courses related to BCI system development and application, such as human-computer interaction, assistive technologies, and neural and rehabilitation engineering, are also part of the curriculum.

**TABLE 4:** Proposed core courses for the BCI major.

Course	Necessity
Brain signal processing	Brain signal processing constitutes a pivotal component of the BCI system and serves as the foundation for effective decoding. Develop students’ ability to create innovative decoding algorithms through hands-on projects (e.g., classification of motor imagery signals).
Principles of BCI	BCI principles is the core course for the BCI major, including BCI theory, principles and methods, and provides a foundation for subsequent courses and practices.
Principles and applications of neuroscience	Neuroscience is the scientific principle of BCI and involves the generation of distinguishable brain signal features that characterize user intent and central neural plasticity, contributing to the innovation of the BCI paradigm and neural coding.
Foundations of cognitive neuroscience	A comprehensive understanding of the neural mechanisms underlying cognitive processes in the brain is essential for the development of cognitive-based BCI systems and the expansion of BCI application areas.
Experimental psychology and BCI paradigm	The development and use of the BCI system are closely related to psychology, supporting the design of experiments for individual behavioral and psychological research, innovating and optimizing the design of the BCI paradigm.
Behavioral psychology for BCI	Gain insights into BCI user behavior and psychological responses to enhance the human-computer interaction efficiency of BCI systems, and enable effective monitoring of user states in various application scenarios.
Principles and practice of neurofeedback	Neurofeedback systems are not only a significant category of BCI but also serve as a crucial component of BCI systems. Neurofeedback training and modulation play a vital role in enhancing the overall performance of BCI systems.
Neural engineering and rehabilitation engineering	The development and application of the BCI system involve the basic principles and methods of neural engineering and rehabilitation engineering. Consequently, a comprehensive understanding of these engineering disciplines is imperative for students pursuing a degree in BCI.
Human-computer interaction	BCI represents a specialized and advanced form of HCI technology. Design principles from HCI, such as optimizing user experience, improving interaction efficiency, and conducting usability evaluations, provide critical guidance for refining interface design, signal feedback mechanisms, and interaction models in BCI systems.
Assistive technology	A significant objective of BCI is to furnish assistive technology for individuals with severe movement disorders and disabilities. The development of BCI is inextricably associated with assistive technology design principles, such as adaptability, accessibility, and usability.
Frontiers in BCI lectures	Track advancements in frontier technologies, cultivate interdisciplinary cognitive frameworks, and enhance adaptability to meet evolving academic and industrial requirements.

From the above, it is evident that the curriculum design for the BCI major, based on an interdisciplinary collaboration for BCI development, includes courses from various academic disciplines. While ensuring that students acquire the necessary foundational knowledge and essential skills closely related to BCI, it is also critical to identify and prioritize core courses in key disciplines.

In curriculum design, foundational courses, core courses, and practical training collectively form a comprehensive knowledge system through progressive layering and interdisciplinary collaboration. Foundational courses provide mathematical and engineering tools for BCI technology. For instance, probability and statistics lay the groundwork for modeling noise in brain signals, linear algebra underpins the matrix operations necessary for feature extraction in machine learning, and digital signal processing algorithms are directly applied to filtering and denoising EEG signals. Core courses (e.g., brain signal processing, human-computer interaction, and neural engineering) focus on cultivating the essential skills of BCI by bridging foundational knowledge with industry requirements through both theoretical deepening and practical application (e.g., developing algorithms for classifying motor imagery-related brain signals and optimizing user interfaces). Practical training, incorporating BCI experiments and industry partnership projects, further integrates interdisciplinary knowledge. This three-tiered framework, which spans from fundamental principles to technological integration and finally to innovative applications, serves to enhance the logical coherence among courses. Moreover, it establishes a closed-loop competency development system through interdisciplinary collaboration (from mathematical modeling to algorithm implementation to clinical validation), ensuring that students systematically master the skills required, from brain signal decoding to system optimization.

## 4 Discussion

### 4.1 What are the challenges in constructing the brain-computer interface major?

There are many challenges in constructing the BCI major, particularly because of its highly multidisciplinary nature, its status as a frontier research area, and its rapid pace of development. Below are some key challenges and suggested strategies for addressing them.

#### 4.1.1 The lack of consensus and international collaboration in the construction of the brain-computer interface major

The BCI major is an emerging major, and there is currently no consensus on how to construct this major especially regarding curriculum design. This lack of consensus results in limited references for major construction. Therefore, international collaboration within the BCI community is essential. Drawing on the advanced experiences of BCI research and education in different regions or countries can help to promote cooperation in constructing the BCI major. In particular, the establishment of an internationally oriented BCI curriculum based on its multidisciplinary nature is crucial. The BCI community has already published several significant works on the topic (Allison et al., 2012; Collura, 2014; Graimann et al., 2010; Ramsey and Millán, 2020; Wolpaw and Wolpaw, 2012).

Existing literature has preliminarily discussed the framework for a BCI major, including the academic system, degree awards, educational objectives, curriculum design, and credit distribution (Yang et al., 2024). However, this literature does not provide detailed syllabi for each course (including learning objectives, content, and teaching methods). This omission may be partly due to space limitations and partly because of the differences in syllabi across regions or countries. For course syllabi, the selection of teaching content is critical. Only the essential content should be included, avoiding the inclusion of all possible topics or a simple compilation of existing materials. Additionally, the literature may have overlooked certain courses that are essential for the BCI major, such as Human-Computer Interaction and Assistive Technology. Finally, aspects such as the prerequisites for each course (i.e., the logical sequence of courses), the objectives of each course and their alignment with the overall educational goals of the major, teaching content, assessment methods, textbooks, and reference materials all require careful consideration.

#### 4.1.2 Inadequate or lack of course materials for brain-computer interface major

As an emerging major, the BCI major is still in its infancy and developmental stages. At present, and for the foreseeable future, there remains a lack of comprehensive or specialized textbooks for its curriculum (Yang et al., 2024). Although the BCI community has already published several significant works (Allison et al., 2012; Collura, 2014; Graimann et al., 2010; Ramsey and Millán, 2020; Wolpaw and Wolpaw, 2012), the development of textbooks for BCI courses still faces the following main challenges.

##### 4.1.2.1 Mismatch between existing brain-computer interface books and the demands of the brain-computer interface curriculum

Existing edited volumes or monographs on BCI primarily focus on research and academics. They often summarize and integrate the work of prominent international research teams in the BCI field. While these are valuable and rich as reference materials for BCI teaching, they may not be entirely suitable as textbooks. It is recommended to fully consider teaching objectives, methods, and principles, organizing the content in a progressive and logical sequence. Although BCI theories, principles, and methods have evolved into a relatively stable and widely accepted framework over the past 50 years, existing BCI books were published at specific points in time and may need to be updated to reflect the latest advances in BCI research. In addition, these publications often lack thought-provoking questions and exercises designed to engage students and inspire innovation.

##### 4.1.2.2 Lack of brain-computer interface experimental material

BCI is rooted in experimental science and technology research as opposed to purely theoretical research. As previously mentioned, the BCI major not only focuses on teaching BCI theories, principles, and methods but also emphasizes the combination of theory and practice through experimental teaching. However, there is currently a lack of complementary experimental manuals tailored for BCI education.

It is advisable to consider incorporating the three classical BCI paradigms (SSVEP-BCI, MI-BCI, and P300-BCI) as the core experimental teaching content, while also adding some of the latest BCI paradigms. For each BCI paradigm in experimental tutorials, the content could include the following: Experimental objectives, required hardware and software, Experimental design, Step-by-step procedures, Full-system implementation and Provide support for computer technology in BCI systems, to understand the fundamentals of computer systems, and to support the design and optimization of BCI system. Developing such experimental manuals will be essential for fostering hands-on skills and practical understanding among students.

##### 4.1.2.3 How to select, integrate, and develop systematic textbooks from multidisciplinary content closely related to brain-computer interface

As previously mentioned, BCI involves multiple disciplines, including neuroscience, psychology and cognitive science, computer science and technology, signal processing and pattern recognition, machine learning and artificial intelligence, human factors engineering, and neurorehabilitation engineering. Based on existing textbooks in these disciplines, it is challenging to determine what to include, omit, or integrate in a systematic BCI textbook. This challenge is particularly significant in ensuring that the content covers the foundational knowledge required for BCI research and development. The design of a BCI curriculum must balance breadth and depth within the constraints of limited class time. It is essential to avoid merely combining materials or attempting to cover everything comprehensively. Instead, a selective and focused approach is recommended. To achieve this, a multidisciplinary team of experts from related fields should collaborate to develop the textbook, with each expert contributing content from their area of expertise while ensuring coherence and integration across disciplines.

##### 4.1.2.4 How to ensure the brain-computer interface curriculum reflects the rapid advancements in brain-computer interface technology

BCI technology is advancing rapidly as neuroscientific, hardware, algorithmic, and applicational advances continue to be made. In particular, advancements in brain signal acquisition technologies, new processing algorithms, machine learning and deep learning, and brain-machine interfaces are occurring at a fast pace. This necessitates frequent adjustments to course content to keep up with the latest developments.

It is recommended to create a “stable edition” textbook that covers the basics, core principles, and methodologies, while periodically updating course content to reflect new technologies and research findings. For example: Introduce elective courses and special-topic seminars to include emerging technologies and the latest research. Supplement textbooks with online resources and up-to-date scientific literature. Periodically update or release digital chapters to facilitate content adjustments in response to advancements. Additionally, students should be encouraged to participate in research projects and academic competitions to deepen their understanding of frontier technologies. This dynamic approach will ensure that the BCI curriculum remains relevant and aligned with the rapid progress in the field.

#### 4.1.3 High-cost brain-computer interface experimental equipment and resources

The construction of BCI as an academic discipline requires the establishment of teaching-oriented BCI laboratories. Most existing BCI laboratories are research-focused rather than tailored for teaching purposes at present. In addition, the BCI hardware and software systems available in the commercial market are generally costly. Given that teaching usually demands multiple experimental platforms, this consequently results in high configuration expenses.

It is recommended to develop BCI experimental systems specifically designed for teaching with significant cost and price reductions to support bulk deployment. In addition, university-industry collaborations and resource-sharing initiatives can address the shortage of experimental facilities. The establishment of virtual simulation platforms for experiments can also help students to simulate experimental procedures and data analysis via software, thus reducing costs (Yang et al., 2024).

#### 4.1.4 Shortage of faculty in the brain-computer interface major

Faculty for the BCI major needs to have a multidisciplinary background. However, there is currently a shortage of full-time faculty in universities with expertise in both neuroscience and computer technology, which impacts the quality of teaching. It is proposed to invite experts with multidisciplinary BCI backgrounds to serve as guest lecturers or mentors. Existing faculty members should be encouraged to participate in multidisciplinary BCI training programs to enhance their expertise and contribute to high-quality teaching at the same time (Yang et al., 2024).

### 4.2 What is the prospect of the BCI major?

The prospects of the BCI major are closely tied to the development of BCI technology and its transition into practical applications. With continuous advancements in fields such as neuroscience, psychology and cognitive science, computer science and technology, artificial intelligence, and ergonomics, BCI technology is expected to move gradually from the laboratory to practical applications. Its use cases will expand beyond medical rehabilitation to encompass education, entertainment, intelligent control, augmented reality, and other domains.

In medical applications, BCI technology will provide new diagnostic, therapeutic, and rehabilitative solutions for individuals with severe motor impairments and disabilities, such as paralyzed patients, individuals with aphasia, and those with other neurological or psychiatric conditions. In non-medical applications, such as brain-controlled games and brain-machine collaborative robotics, BCI technology will bring novel experiences and services to healthy individuals. These applications will provide diverse industries for BCI students to showcase their talents.

### 4.3 Limitations of the study and future work

Based on the multidisciplinary nature of BCI technology, this paper proposes eight principles for designing the BCI curriculum and develops the hierarchical structure of curriculum design for the BCI major that includes basic courses in discipline, introductory courses, core courses of the major, practice training, and comprehensive training for dissertations. This hierarchical structure provides a reference for the construction of the BCI major. However, the guidelines for designing the BCI curriculum still require in-depth consideration, supplementation, and refinement; it is especially necessary to evaluate the curriculum design of the BCI major from a competency-oriented perspective, which may include the following:

(1) Clearly defining the skills and knowledge covered by the BCI major. This includes supporting evidence of these skills and knowledge through needs assessment studies, interviews, analysis of future jobs, market research, job positions, etc.
(2) Matching the skills and knowledge with the appropriate teaching methods and techniques.
(3) Designing a structured curriculum grid. The grid can provide insights into how to weave and interleave the skills and knowledge across different courses, including the workload of classroom and independent assignments.
(4) Integrating ethical issues related to BCI throughout the curriculum. Because of the direct connection between BCI and the medical practice and the human brain, Ethical issues should be covered across the curriculum, with putting the human in the center rather than the technology.

## 5 Conclusion

This paper focuses on the emerging BCI major and based on the inherent and essential multidisciplinary nature of BCI system research and development, proposes curriculum design principles for the BCI major and provides recommendations for its curriculum structure. These principles and recommendations are preliminary. The next step is to improve the BCI major’s curriculum design from a competency-oriented perspective. As BCI technology continues to evolve, the curriculum design and development of the BCI major will be further refined. The challenges in the construction of the BCI major will be gradually overcome, paving the way for a brighter future for this major.
